# Middle-Range theory of the nursing diagnosis of sedentary lifestyle in young adults

**DOI:** 10.1590/0034-7167-2023-0516

**Published:** 2024-09-06

**Authors:** Renata Marinho Fernandes, Ana Carolina Costa Carino, Anna Thays Dias Almeida, Maria Isabel da Conceição Dias Fernandes, Sâmella Karine de Macêdo Leopoldino, Camila Sayonara Tavares Gomes, Ana Raquel Cortês Nelson, Ana Luisa Brandão de Carvalho Lira

**Affiliations:** I Universidade Federal do Rio Grande do Norte. Natal, Rio Grande do Norte, Brazil; II Universidade Federal do Ceará. Ceará, Fortaleza, Brazil

**Keywords:** Nursing Diagnosis, Nursing Theory, Sedentary Behavior, Young Adult, Health Promotion, Diagnóstico de Enfermería, Teoría de Enfermería, Conducta Sedentaria, Adulto Joven, Promoción de la Salud

## Abstract

**Objective:**

To construct a middle-range theory for the nursing diagnosis of Sedentary Lifestyle in young adults.

**Methods:**

A methodological study for the validation of a nursing diagnosis based on a Middle-Range Theory, carried out in six stages: definition of the approach; definition of theoretical-conceptual models; definition of main concepts; development of a pictorial scheme; construction of propositions; establishment of causal relationships and evidence for practice. The theory construction was operationalized through an integrative review and supported by Roy’s adaptation model.

**Results:**

Three essential attributes were identified; 10 antecedents; 7 clinical consequences; a pictogram, 9 propositions, and 11 causal relationships and evidence for practice.

**Conclusion:**

The middle-range theory for the nursing diagnosis of Sedentary Lifestyle in young adults was constructed, expanding the understanding of this phenomenon, to be applied in clinical practice by nurses.

## RESUMEN

**Objetivo:**

Construir una teoria de alcance medio para el diagnóstico de enfermería de Estilo de Vida Sedentario en jóvenes adultos.

**Métodos:**

Un estudio metodológico para la validación de un diagnóstico de enfermería basado en una Teoría de Alcance Medio, llevado a cabo en seis etapas: definición del enfoque; definición de modelos teórico-conceptuales; definición de conceptos principales; desarrollo de un esquema pictórico; construcción de proposiciones; establecimiento de relaciones causales y evidencia para la práctica. La construcción de la teoría se operacionalizó a través de una revisión integrativa y se sustentó en el modelo de adaptación de Roy.

**Resultados:**

Se identificaron tres atributos esenciales; 10 antecedentes; 7 consecuencias clínicas; un pictograma, 9 proposiciones y 11 relaciones causales y evidencia para la práctica.

**Conclusion:**

Se construyó la teoría de alcance medio para el diagnóstico de enfermería de Estilo de Vida Sedentario en jóvenes adultos, ampliando la comprensión de este fenómeno, para ser aplicado en la práctica clínica por los enfermeros.

## INTRODUCTION

Sedentary behavior contributes to the increased prevalence of chronic diseases^(1)^. Approximately 1.4 billion adults are at risk of developing or exacerbating conditions related to physical inactivity^(2)^. Sedentary behavior is associated with higher all-cause mortality rates^(3)^.

In this context, the literature highlights the need to individualize and adapt lifestyle recommendations to have a real impact on people’s health^(3)^. The World Health Organization (WHO), through its Guidelines on Physical Activity and Sedentary Behavior, also emphasizes a lack of research involving specific populations^(4)^.

Sedentary lifestyle is defined in nursing as an acquired behavior pattern characterized by activities during wakefulness that require low energy expenditure^(5)^. However, this definition is not directed toward a specific audience, such as children, adolescents, adults, or the elderly.

Furthermore, in 2020, with the COVID-19 pandemic, cases of sedentary behavior increased significantly due to the confinement imposed by public health authorities^(6)^. In this scenario, studies conducted among young adults indicated a significant decrease in physical activity levels. A prevalence of 65.2% of sedentary behavior in young adults was identified, along with a 70% reduction in hours spent on physical activities^(7, 8)^.

Therefore, it is evident that sedentary behavior is present in the clinical practice of nurses and, for this reason, needs to be researched to recognize its attributes^(9)^. In this interim, the development of middle-range theories (MRT) aims to bridge the gap between theory and practice, mainly through knowledge derived from research^(10)^. MRT involves understanding nursing phenomena through less abstract concepts and propositions, contributing to the profession’s development^(11)^.

Among international taxonomies on nursing diagnoses (ND), NANDA International stands out, presenting Sedentary Lifestyle (00168) in its diagnostic scope, with a level of evidence of 3.3^(5)^. However, no MRT on this diagnosis in young adults has been identified in the literature to date. Only MRT related to nursing diagnoses such as Ineffective Infant Feeding Pattern^(12)^, Low Health Self-Efficacy^(13)^, Dysfunctional Ventilatory Weaning Response^(14)^, and Excess Fluid Volume^(15)^ were found.

In this context, it is emphasized that MRTs have been considered highly applicable in research and clinical practice. However, more studies on the subject are needed^(11)^, specifically studies on MRT for the nursing diagnosis of Sedentary Lifestyle aimed at the young adult population.

Given the above and in the pursuit of strengthening knowledge and improving nursing care directed at this clientele, the present study is proposed based on the hypothesis that the nursing diagnosis of Sedentary Lifestyle in young adults has particularities different from other age groups.

Therefore, the study aims to construct a middle-range theory for the nursing diagnosis of Sedentary Lifestyle in young adults. It is expected to contribute to the advancement of knowledge on this phenomenon and thus facilitate its early identification in clinical practice, as well as strengthen nursing as a profession and science.

## OBJECTIVE

To construct a middle-range theory for the nursing diagnosis of Sedentary Lifestyle in young adults.

## METHODS

### Ethical Aspects

This research is theoretical in nature, and the copyrights of the publications included in the research were respected. Therefore, it complied with Law No. 9,610, of February 19, 1998, which amends, updates, and consolidates copyright legislation and provides other provisions^(16)^.

### Type of Study

This is a methodological study for the validation of a nursing diagnosis based on the development of a Middle-Range Theory (MRT), grounded in the theoretical-causal validity framework of Lopes, Silva & Herdman^(17)^. The study was conducted in six stages: definition of the approach for constructing the middle-range theory; definition of the theoretical-conceptual models to be analyzed; definition of the main concepts of the MRT; development of a pictorial scheme; construction of the MRT propositions; and establishment of causal and evidence-based relationships for practice.

In the first stage, the primary goals of this MRT for the nursing diagnosis of Sedentary Lifestyle in young adults are to establish precise relationships between concepts, aiming to describe how changes occur within a phenomenon. According to the literature^(17)^, these characteristics are present in predictive theory. Thus, this theory is predictive.

MRTs can follow inductive, deductive, or both orientations^(11)^. The present MRT followed a deductive orientation, as it was constructed based on the results of an integrative literature review. This research was conducted to identify the common elements of the Sedentary Lifestyle diagnosis (00168) and its conceptual and operational definitions.

The stage related to defining the theoretical-conceptual models to be analyzed will result in the development of the basic structure of the future MRT^(17)^. Thus, the theoretical model proposed by Roy, which offers conceptions about the person’s adaptive system and its stimuli, was adopted. In this theoretical model, the person is seen as a system that can be affected by the environment or stimuli. These influencing factors are subdivided into: focal, contextual, and residual stimuli. The focal stimulus represents what immediately confronts a person, causing the greatest impact. Contextual stimuli are those that influence the effect of the focal stimulus. Finally, residual stimuli produce non-central effects; generally, the person is not aware of these stimuli’s existence, nor is it clear to the observer the effect produced by this type of stimulus^(18)^.

These conceptions were combined with the results of the literature review, the elements that compose clinical reasoning models, the process of validating nursing diagnoses, and epidemiological causal models.

Thus, for the operationalization of the integrative literature review, the following guiding question was established: What are the clinical indicators and causal factors of a Sedentary Lifestyle in young adults? The recommendations of the Preferred Reporting Items for Systematic Reviews and Meta-Analyses (PRISMA) were adapted as applicable to this review^(19)^.

### Methodological Procedures

#### Data Collection, Organization, and Analysis

Data collection occurred between August and October 2020, in the following databases: National Library of Medicine and National Institutes of Health (PubMed); Scopus; Cumulative Index to Nursing and Allied Health Literature (CINAHL); and Web of Science. The material search was conducted through the CAPES Periodicals Portal, using the Federated Academic Community (CAFe) of the Federal University of Rio Grande do Norte.

Four Medical Subject Headings (MESH) descriptors were used: “Sedentary Behavior”; “Sedentary Lifestyle”; “Young Adult”; and “Adolescent.” The search strategy was outlined using the Boolean operators AND and OR, as follows: ((“Sedentary Behavior” OR “Sedentary Lifestyle”) AND (“Young Adult” OR “Adolescent”)). The timeframe was limited to 5 years (2016-2020) to find the most recent studies on the topic and to facilitate the review process, as described in the literature^(20, 21)^.

The inclusion criteria were: complete articles available in full text; in Portuguese, English, or Spanish; studies involving populations aged 18 to 24 years. Exclusion criteria included: studies in the format of editorials; protocols; letters to the editor; abstracts; and literature reviews.

**Figure 1:**
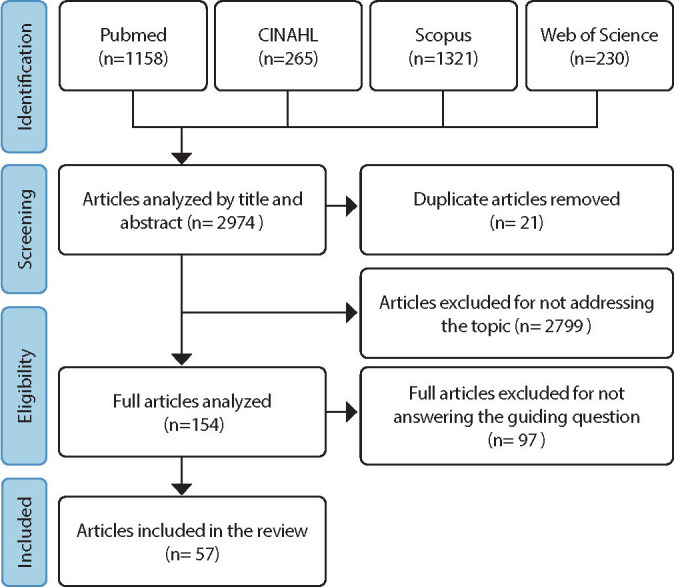
Flowchart of the search and selection process in each database. Natal, Brazil, 2022

After reaching a consensus, a final sample of 57 articles was obtained. The flowchart below (Figure 1) presents the search and selection process in each database.

After conducting the integrative review, the contents were analyzed, and the three main concepts for constructing the MRT (essential attributes, clinical antecedents, and clinical consequences) were extracted from the articles by the principal researcher. According to the literature^(17)^, attributes are key elements that define the diagnosis. Clinical antecedents are etiological factors, the causes of the situation, and clinical consequences are the signs and symptoms. These components were defined conceptually and operationally by the principal researcher from November to December 2020.

For the analysis of the level of evidence of each study, the recommendations of the JBI were followed to characterize the studies identified for the sample. The articles were categorized by levels of evidence of effectiveness, ranging within five distinct levels, from opinion studies to randomized clinical trials^(22)^. No study was excluded at this stage.

A pictogram was created to facilitate the understanding of the relationship between essential attributes, clinical antecedents, and clinical consequences in the studied population. Next, propositions for the ND Sedentary Lifestyle in young adults were developed. Finally, the causal relationships between etiological factors (clinical antecedents) and the sedentary lifestyle were established. This stage aims to improve the clinical use of the ND by providing evidence for nursing practice^(17)^.

## RESULTS

### Definition of the Approach

The approach chosen for constructing the middle-range theory was the integrative literature review, as suggested by the theoretical-causal validity framework of Lopes, Silva & Herdman^(17)^. A total of 57 articles were selected to form the sample, with the highest frequency of publication occurring in 2019 (33.3%). Most of the studies presented a level of evidence of 4.b (cross-sectional study) (54.3%) and were published in English (89.4%)^(23)^.

### Main Concepts (Key Concepts)

The essential attributes identified were: personal and social characteristics; physical inactivity; and an average level of physical activity below the recommended amount. Subsequently, the essential attributes were grouped by the principal researcher, and the definition of the diagnosis under study was constructed: “Personal and social characteristics and physical inactivity or physical activity below the recommended level.”

The clinical antecedents, also referred to as etiological factors, were: advancing age; deficient knowledge about sedentarism; unemployment; high socioeconomic status; unfavorable personal and family habits regarding physical activity; pollution and unfavorable environmental conditions for physical activity; female gender; living in urban areas; excessive screen time; and prolonged sitting time.

Regarding clinical antecedents (etiological factors), these were categorized according to Roy’s theoretical model into focal, contextual, and residual stimuli. The focal stimuli are: unfavorable personal and family habits regarding physical activity; excessive screen time; and prolonged sitting time. The contextual stimuli are: deficient knowledge about sedentarism; and pollution and unfavorable environmental conditions for physical activity. The residual stimuli are: advancing age; unemployment; high socioeconomic status; female gender; and living in urban areas.

The clinical consequences were: cardiovascular changes; excess adiposity; lack of physical conditioning; impaired cognitive function; low back pain; impaired mental health; and inadequate sleep duration.

### Pictorial Scheme (Pictogram)

**Figure 2:**
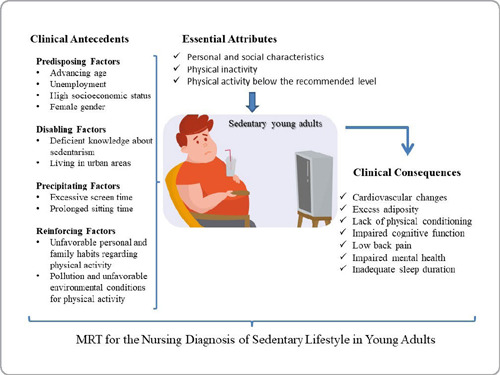
Pictorial scheme of the middle-range theory for the nursing diagnosis of Sedentary Lifestyle in young adults. Brazil, 2022

The clinical antecedents were classified into predisposing factors, disabling factors, precipitating factors, and reinforcing factors. The literature^(17)^ considers a predisposing factor as one that creates a status of susceptibility, a disabling factor as one that interferes with recovery or health promotion, a precipitating factor initiates the causal chain, and a reinforcing factor amplifies the effect of an existing condition. This division will be presented in the proposed pictogram (Figure 2).

### Propositions

The propositions developed in the present MRT are:

1. Personal and environmental stimuli are related to the adoption of sedentary behaviors.
2. Female individuals have a tendency to develop a sedentary lifestyle.
3. Factors related to technological advances, such as the internet and smartphones, contribute to the adoption of a sedentary lifestyle.
4. A sedentary lifestyle in young adulthood can cause mental health issues, such as depression, anxiety, and high levels of stress.
5. Insufficient sleep is common among sedentary individuals.
6. A sedentary lifestyle is related to an increased risk of developing cardiovascular changes in young adults.
7. A sedentary lifestyle affects the daily activities of young adults, particularly in work and study, due to the impact on memory and cognitive function.
8. Sedentary behavior can cause low back pain in this age group.
9. A sedentary lifestyle in young adults contributes to increased adiposity and decreased physical conditioning.

### Causal Relationships and Evidence for Practice

The causal relationships between the etiological factors of the ND Sedentary Lifestyle in young adults are:

1. Advancing Age: The aging process causes transitions, notably moving away from home and starting university or employment. These transitions lead to physical, behavioral, and interpersonal changes that can be linked to sedentary behavior^(24, 25)^.
2. Unemployment: Unemployment can cause sedentary behaviors because jobs in youth generally involve physical activities and standing positions. Therefore, regular physical activity is more common^(26)^.
3. Female Gender: Due to cultural reasons and/or changes from puberty, such as increased fat deposits, discomfort associated with regular menstrual cycles, and reduced hemoglobin levels in the blood, among others, females are more likely to adopt a sedentary lifestyle^(27, 28)^.
4. Deficient Knowledge about Sedentarism: Individuals who lack knowledge about the harms of sedentary behavior and, consequently, the benefits of regular physical activity and healthy lifestyle adoption may develop sedentary behavior^(29)^.
5. Living in Urban Areas: Residing in urban areas is associated with lower physical activity levels due to work styles and life routines in urban centers^(30)^.
6. Excessive Screen Time: Excessive screen time (>2h) is related to sedentarism, as it typically involves positions that require little energy expenditure^(26)^.
7. Prolonged Sitting Time: Long periods spent sitting, on average 7 hours/day, whether studying, playing games, or watching television, are sedentary behaviors^(31)^.
8. Unfavorable Personal and Family Habits for Physical Activity: The absence or insufficiency of physical activity and/or healthy behaviors in families leads to a greater disinterest in young adults in exercising^(32, 33)^.
9. Pollution and Unfavorable Environmental Conditions for Physical Activity: Pollution, rain, extreme heat or cold can cause discomfort, fatigue, and drowsiness, leading to a tendency toward sedentary behavior^(29, 34)^.

It is essential to emphasize the theoretical foundation that nurses need to understand the real causes of a sedentary lifestyle in young adults. This understanding enables them to take health promotion actions considering all the particularities of this population. The MRT emerges as a unique tool for bridging the gap between theory and practice, making the nursing process more accurate and, therefore, more effective.

## DISCUSSION

A sedentary lifestyle manifests itself in specific ways across different age groups. This health behavior is strongly related to technological advancements. The convenience and comfort provided by modernity induce the practice of sedentary habits, especially among young adults^(34)^.

Personal and social characteristics, such as being a student, having a mother with higher education, and having a diminished self-perception of health, are essential attributes for the presence of the nursing diagnosis Sedentary Lifestyle in this population. Studies show that these factors have a significant impact on the adoption of physical exercise practices. These factors are related to the environment, culture, and socioeconomic system, which induce a sedentary lifestyle^(35)^.

Furthermore, physical activity that is less than recommended and physical inactivity are strongly related to changes that occur with aging, the beginning of adulthood, professional activities, or unemployment^(36, 37)^. The changes that occur at this stage of life can interfere with the health care actions adopted by young adults.

The deficient knowledge of this population about a sedentary lifestyle can be evidenced in Campos’ study^(38)^, which pointed out that young adults with more information about the benefits of regular exercise are more physically active, and this is inversely proportional to the lack of such awareness. Moreover, the female gender showed a strong relationship with the adoption of a sedentary lifestyle. Data corroborating previous studies reaffirm higher prevalence of sedentarism in women in this age group^(34, 39)^.

It is also noted that the lack or low frequency of physical activity is related to various health problems among women^(40)^. A cohort study conducted in Brazil identified that young women were more inactive and had a higher percentage of body fat^(41)^.

Another important finding in the present study reveals that young adults living in urban centers engage in less physical activity. A study conducted with families of overweight and sedentary young adults found that 72% of the participants lived in large urban centers^(35)^.

As clinical consequences of the ND Sedentary Lifestyle, cardiovascular diseases and impaired mental health were observed. A study associated sedentarism with increased cardiovascular risks in young adults, as well as with some psychosocial factors such as stress, anxiety, and depression^(42)^. Another study showed that the habit of regular physical exercise improves the psychological aspects and mental health of individuals^(43)^.

Musculoskeletal inaptitude and low back pain are also clinical consequences of the ND under study. Physical conditioning contributes to cardiorespiratory capacity, body composition, muscular endurance, and posture. Moreover, regular physical activity, considering the type of exercise, level, and age, can help prevent low back pain^(44)^. Low back pain is present in the young adult population, related, among other causes, to a sedentary lifestyle^(45)^.

There is also an association between excessive screen time and inadequate sleep. Young adults who spend long periods in sedentary behaviors may experience a decrease in sleep quality^(46)^. Additionally, the cognitive function and memory of this population may be impaired. Research indicates that physical activity increases neurotransmitter levels and improves brain function, as well as cognitive systems and memory processes^(47)^. Therefore, the intellectual performance of these young adults may be affected by adopting this behavior.

Thus, authors encourage the development of interventions that promote the health of this population, aiming at their well-being and quality of life^(34)^. The adoption of a sedentary lifestyle in young adults is concerning and has particularities that should be considered in future recommendations proposed by the WHO.

### Limitations of the Study

A limitation of the study is the restriction of the search to English, Portuguese, and Spanish, as articles in other languages could have addressed the research question. Another limitation is the extraction of essential attributes, antecedents, and clinical consequences performed solely by the principal researcher, without the assistance of other researchers and/or software.

Additionally, there is a limitation related to the lack of testing of the present theory in clinical practice. Therefore, the development of studies to empirically validate this MRT in the population of young adults is encouraged.

### Contributions to the Field

Given the above, the development of MRT contributes to reducing the gap between theory and practice and to strengthening the science of nursing. MRT focused on nursing diagnoses allow for an in-depth understanding of the phenomenon of interest and thus facilitate its early identification in clinical practice by nurses.

The MRT of Sedentary Lifestyle in young adults can be a useful tool, as it broadens the understanding of this phenomenon, its etiological elements, clinical characteristics, and causal relationships. This can contribute to the clinical reasoning and diagnostic judgment of nurses in setting goals for protection, promotion, and support for the adoption of healthy lifestyles

## CONCLUSION

The theoretical construction identified three essential attributes: personal and social characteristics, physical inactivity, and an average level of physical activity below the recommended amount. Additionally, 10 clinical antecedents and 7 clinical consequences were identified. Among these, 3 clinical antecedents were classified as focal stimuli, 2 as contextual stimuli, and 5 as residual stimuli. Furthermore, a pictogram, 9 propositions, and 11 causal relationships and evidence for practice derived from the interrelations between these concepts were developed. These steps were essential for a better understanding of the studied diagnosis.

Thus, this theoretical construction is timely and produces tools that can assist in promoting the health and quality of life of this specific population. It will facilitate the early identification by nurses of the phenomenon of Sedentary Lifestyle in young adults, highlighting the following etiological factors: unfavorable personal and family habits regarding physical activity, excessive screen time, prolonged sitting time, and deficient knowledge about sedentarism. These etiological factors were highlighted because they are more directly related to the independent decision-making of nurses and, when managed, can promote healthy behaviors and increase comfort.

Theoretical studies on nursing diagnoses should be conducted with the aim of making nursing care more effective, individualized, and of higher quality, thus strengthening the science of nursing.
