# Supplementary material for: Middle-Range theory of the nursing diagnosis of sedentary lifestyle in young adults
Source: Rev Bras Enferm. 2024 Sep 6;77(4):e20230516. doi: 10.1590/0034-7167-2023-0516 (PMC11382669; doi:10.1590/0034-7167-2023-0516)
Supplement: Translation

# Translation: Teoria de médio alcance do diagnóstico de enfermagem estilo de vida sedentário em jovens adultos

**DOI:** 10.1590/0034-7167-2023-0516pt

**Authors:** Renata Marinho Fernandes, Ana Carolina Costa Carino, Anna Thays Dias Almeida, Maria Isabel da Conceição Dias Fernandes, Sâmella Karine de Macêdo Leopoldino, Camila Sayonara Tavares Gomes, Ana Raquel Cortês Nelson, Ana Luisa Brandão de Carvalho Lira

**Affiliations:** I Universidade Federal do Rio Grande do Norte. Natal, Rio Grande do Norte, Brasil; II Universidade Federal do Ceará. Ceará, Fortaleza, Brasil

**Keywords:** Diagnóstico de Enfermagem, Teoria de Enfermagem, Comportamento Sedentário, Adulto Jovem, Promoção da Saúde

## Abstract

**Objetivo:**

Construir uma teoria de médio alcance para o diagnóstico de enfermagem Estilo de vida sedentário em jovens adultos.

**Métodos:**

Estudo metodológico de validação de diagnóstico de enfermagem a partir de uma Teoria de Médio Alcance, realizado em seis etapas: definição da abordagem; definição dos modelos teórico-conceituais; definição dos conceitos principais; desenvolvimento do esquema pictorial; construção de proposições; estabelecimento das relações de causalidade e de evidência para a prática. A construção da teoria foi operacionalizada por meio de uma revisão integrativa e subsidiada pelo modelo teórico adaptativo de Roy.

**Resultados:**

Foram identificados três atributos essenciais; 10 antecedentes; 7 consequentes clínicos; um pictograma, 9 proposições e 11 relações de causalidade e evidências para a prática.

**Conclusão:**

A teoria de médio alcance para o diagnóstico de enfermagem estilo de vida sedentário em adultos jovens foi construída, ampliando o conhecimento desse fenômeno, a fim de ser aplicado na prática clínica pelo enfermeiro.

## INTRODUÇÃO

O sedentarismo contribui para o aumento da prevalência das doenças crônicas^(1)^. Aproximadamente 1,4 bilhões de adultos correm o risco de desenvolver ou agravar doenças ligadas à inatividade física^(2)^. O comportamento sedentário está associado a maiores índices de mortalidade por todas as causas^(3)^.

Nesse contexto, a literatura evidencia a necessidade de individualizar e adaptar as recomendações de estilo de vida, para que haja real impacto na saúde das pessoas^(3)^. Destaca-se ainda a Organização Mundial da Saúde (OMS), que, por meio das Diretrizes para atividade física e comportamento sedentário, revela uma escassez de pesquisas envolvendo populações específicas^(1)^.

O estilo de vida sedentário é definido, pela enfermagem, como um modo de comportamento adquirido, caracterizado por atividades durante a vigília que requerem baixo consumo de energia^(5)^. Todavia, o mesmo não está direcionado para um público específico, como crianças, adolescentes, adultos ou idosos.

Outrossim, em 2020, com a pandemia da Covid-19, os casos de sedentarismo aumentaram bastante devido ao confinamento imposto pelos órgãos públicos de saúde^(6)^. Nesse cenário, estudos realizados em adultos jovens apontaram uma diminuição significativa nos níveis de atividades físicas realizadas. Foi identificada uma prevalência de 65,2% de comportamento sedentário em jovens adultos e uma queda de 70% nas horas despendidas em atividades físicas^(7, 8)^.

Destarte, tem-se que o comportamento sedentário está presente na prática clínica dos enfermeiros e, por esse motivo, precisa ser pesquisado, para que seus atributos sejam reconhecidos^(9)^. Nesse ínterim, o desenvolvimento de teorias de médio alcance (TMA) tem por objetivo reduzir a lacuna entre teoria e prática, principalmente por meio do conhecimento oriundo de pesquisas^(10)^. A TMA envolve a compreensão dos fenômenos próprios da enfermagem, por meio de conceitos e proposições menos abstratas, contribuindo para o desenvolvimento da profissão^(11)^.

Dentre as taxonomias internacionais sobre diagnósticos de enfermagem (DE), ressalta-se a NANDA Internacional, a qual apresenta o Estilo de Vida Sedentário (00168) em seu escopo diagnóstico, com nível de evidência 3.3^(5)^. Entretanto, nenhuma TMA sobre esse diagnóstico em jovens adultos foi identificada na literatura até o momento. Foram encontradas apenas teorias de médio alcance relacionadas aos diagnósticos de Enfermagem: Padrão ineficaz de alimentação infantil^(12)^, Baixa Autoeficácia em Saúde^(13)^, Resposta disfuncional ao desmame ventilatório^(14)^ e Volume de Líquidos Excessivo^(15)^.

Neste contexto, salienta-se que as TMAs têm sido consideradas de alta aplicabilidade na pesquisa e prática clínica. Contudo, são necessárias mais produções sobre o tema^(11)^, especificamente estudos sobre TMA do diagnóstico de enfermagem Estilo de Vida Sedentário direcionado para a população de jovens adultos.

Frente ao exposto e na busca de fortalecer o conhecimento e aprimorar o cuidado de enfermagem direcionado a essa clientela, o presente estudo é proposto, pois acredita-se na hipótese de que o diagnóstico de Enfermagem Estilo de vida sedentário em jovens adultos possuem particularidades diferentes das demais faixas etárias.

Desse modo, o estudo tem como objetivo construir uma teoria de médio alcance para o diagnóstico de enfermagem Estilo de vida sedentário em jovens adultos. Espera-se, assim, contribuir para o avanço no conhecimento sobre esse fenômeno e, assim, facilitar sua identificação de forma precoce na prática clínica, bem como fortalecer a enfermagem enquanto profissão e ciência.

## OBJETIVO

Construir uma teoria de médio alcance para o diagnóstico de enfermagem Estilo de vida sedentário em jovens adultos.

## MÉTODOS

### Aspectos éticos

Esta pesquisa é de natureza teórica e os direitos autorais das publicações incluídas na pesquisa foram respeitados. Portanto, cumpriu-se a Lei nº 9.610, de 19 de fevereiro de 1998, que altera, atualiza e consolida a legislação de direitos autorais e dá outras providências^(16)^.

### Tipo de estudo

Trata-se de um estudo do tipo metodológico de validação de diagnóstico de enfermagem a partir do desenvolvimento de uma Teoria de Médio Alcance, embasado pelo referencial de validade teórico-causal de Lopes, Silva & Herdman^(17)^. Assim, o estudo ocorreu por meio de seis etapas, a saber: definição da abordagem para construção da teoria de médio alcance; definição dos modelos teórico-conceituais a serem analisados; definição dos conceitos principais da TMA; desenvolvimento de um esquema pictórico; construção das proposições da TMA; e estabelecimento das relações de causalidade e de evidência para a prática.

Na primeira etapa, as finalidades elementares da presente TMA para o diagnóstico de enfermagem Estilo de Vida Sedentário em jovens adultos são estabelecer as relações precisas entre os conceitos, visando descrever como ocorrem mudanças dentro de um fenômeno. Segundo a literatura^(17)^, essas características estão presentes na teoria preditiva. Assim, a presente teoria é preditiva.

As TMA podem seguir orientação indutiva, dedutiva ou ambas^(11)^. A presente TMA conjugou a orientação dedutiva, pois foi construída a partir dos resultados de uma revisão integrativa da literatura. Essa pesquisa foi realizada com o intuito de identificar os elementos em comum do diagnóstico Estilo de Vida Sedentário (00168) e suas definições conceituais e operacionais.

A etapa relativa à definição dos modelos teórico-conceituais a serem analisados resultará no processo de desenvolvimento da estrutura básica da futura TMA^(17)^. Dessa forma, partiu-se do modelo teórico proposto por Roy, o qual oferece concepções sobre o sistema adaptativo da pessoa e seus estímulos. Nesse modelo teórico, a pessoa é vista como um sistema que pode ser afetado pelo ambiente ou por estímulos. Esses fatores influenciadores subdividem-se em: estímulos focais, contextuais e residuais. O estímulo focal representa aquele que confronta imediatamente uma pessoa, causando maior impacto nos indivíduos. Os estímulos contextuais são aqueles que influenciam na produção do efeito do estímulo focal. E, por fim, os estímulos residuais produzem efeitos não centrais; geralmente, a pessoa não tem consciência sobre a existência desses estímulos, bem como pode não ser claro para o observador o efeito produzido por esse tipo de estímulo^(18)^.

Somaram-se as essas concepções, os resultados da revisão da literatura, os elementos que compõem modelos de raciocínio clínico, o processo de validação de diagnósticos de enfermagem e os modelos causais epidemiológicos.

Assim, para a operacionalização da revisão integrativa da literatura. Estabeleceu-se como questões norteadora: Quais os indicadores clínicos e fatores causais do Estilo de Vida Sedentário em jovens adultos? As recomendações do *Preferred Reporting Items for Systematic Reviews and Meta-Analyses ‘*Prisma’ foram adaptadas no que contempla esta revisão^(19)^.

### Procedimentos metodológicos

#### Coleta, organização e análise dos dados

A coleta de dados ocorreu entre os meses de agosto e outubro de 2020, nas bases de dados: *National Library of Medicine and National Institutes of Health (PubMed); Scopus; Cumulative Index to Nursing and Allied Health Literature (CINAHL);* e *Web of Science.* A busca dos materiais ocorreu por meio do Portal de Periódicos da CAPES, a partir da Comunidade Acadêmica Federada (CAFe) da Universidade Federal do Rio Grande do Norte.

Foram adotados quatro descritores do *Medical Subject Headings* (MESH): “*Sedentary Behavior”; “Sedentary Lifestyle”; “Young Adult”; “Adolescent”.* A estratégia de busca foi traçada com o auxílio dos operadores booleanos AND e OR, a saber: *((“Sedentary Behavior” OR “SedentaryLifestyle”*) *AND* (“*Young Adult” OR “Adolescent”*)). Delimitou-se a temporalidade de 5 anos (2016-2020), com a finalidade de encontrar estudos mais recentes sobre a temática, e para viabilizar o andamento da revisão, conforme descrito na literatura^(20, 21)^.

Como critérios de inclusão, estabeleceram-se: artigos completos disponíveis na íntegra; nos idiomas português, inglês ou espanhol; estudos envolvendo população com idade entre 18 a 24 anos. Foram excluídos: estudos em formato de editorial; protocolos; cartas ao editor; resumos; e revisões de literatura.

Após consenso, obteve-se uma amostra final de 57 artigos. O fluxograma a seguir (Figura 1) apresenta o processo de busca e seleção nas bases de dados.

**Figura 1:** Fluxograma do processo de busca e seleção em cada base de dados. Brasil, 2022

Após a realização da revisão integrativa, os conteúdos foram analisados e os três conceitos principais para a construção da TMA (atributos essenciais, antecedentes clínicos e consequentes clínicos) foram extraídos dos artigos pela pesquisadora principal. Segundo a literatura^(17)^, atributos são elementos-chave que definem o diagnóstico. Antecedentes clínicos são fatores etiológicos, os causadores da situação, e consequentes clínicos são os sinais e sintomas clínicos. Esses componentes foram definidos conceitualmente e operacionalmente pela pesquisadora principal, no período de novembro a dezembro de 2020.

Para a análise do nível de evidência de cada estudo, seguiram-se as recomendações do JBI, com o intuito de caracterizar os estudos identificados para compor a amostra. Os artigos foram categorizados mediante níveis de evidência de eficácia, que variam dentro de cinco níveis distintos, desde estudos de opinião até ensaios clínicos randomizados^(22)^. Nenhum estudo foi excluído nessa etapa.

Foi elaborado um pictograma para facilitar a compreensão da relação entre os atributos essenciais, os antecedentes clínicos e os consequentes clínicos na população estudada. Em seguida, foram desenvolvidas proposições do DE Estilo de Vida Sedentário em jovens adultos. Por último, foram estabelecidas as relações de causalidade entre os fatores etiológicos (antecedentes clínicos) e o estilo de vida sedentário. Essa etapa tem como finalidade melhorar a compreensão do uso clínico do DE, trazendo evidências para a prática de enfermagem^(17)^.

## RESULTADOS

### Definição da abordagem

A abordagem escolhida para a construção da teoria de médio alcance foi a revisão integrativa da literatura, conforme sugere o referencial de validade teórico-causal de Lopes, Silva & Herdman^(17)^. Selecionou-se 57 artigos para compor a amostra, ocorrendo a maior frequência de publicação no ano de 2019 (33,3%). A maioria do estudo apresentou o nível de evidência 4.b (estudo transversal) (54,3%) e foram publicados em língua inglesa (89,4%)^(23)^.

### Conceitos principais (conceitos-chave)

Os atributos essenciais identificados foram: características pessoais e sociais; inatividade física e média de atividade física menor que a recomendada. Em seguida, os atributos essenciais foram agrupados pela pesquisadora principal, e construiu-se a definição do diagnóstico em estudo: “Características pessoais e sociais e inatividade física ou atividade física menor que a recomendada.”

Os antecedentes clínicos, também denominados fatores etiológicos, foram: avançar da idade;

conhecimento deficiente sobre sedentarismo; desemprego; elevado nível socioeconômico; hábitos pessoais e familiares desfavoráveis para a atividade física; poluição e condições ambientais desfavoráveis para a atividade física; sexo feminino; morar em zona urbana; tempo de tela excessivo; e, tempo sentado prolongado.

Ainda em relação aos antecedentes clínicos (fatores etiológicos), esses foram categorizados conforme o modelo teórico de Roy em estímulos focais, contextuais e residuais. Assim, os estímulos focais são: hábitos pessoais e familiares desfavoráveis para a atividade física; tempo de tela excessivo; e, tempo sentado prolongado. Os estímulos contextuais são: conhecimento deficiente sobre sedentarismo; e, poluição e condições ambientais desfavoráveis para a atividade física. Os estímulos residuais são: avançar da idade; desemprego; elevado nível socioeconômico; sexo feminino; e, morar em zona urbana.

E os consequentes clínicos foram: alterações cardiovasculares; excesso de adiposidade; falta de condicionamento físico; função cognitiva prejudicada; lombalgia; saúde mental prejudicada; e, tempo de sono inadequado.

### Esquema pictorial (pictograma)

Os antecedentes clínicos foram classificados em fatores predisponentes, fatores incapacitantes, fatores precipitantes e fatores de reforço. A literatura^(17)^ considera um fator predisponente aquele que cria um status de suscetibilidade, o fator incapacitante é aquele que interfere na recuperação ou promoção da saúde, o fator precipitante inicia a cadeia causal e o fator de reforço amplifica o efeito de uma condição existente. Essa divisão será apresentada no pictograma proposto (Figura 2).

**Figura 2:** Esquema pictorial da teoria de médio alcance para o diagnóstico de enfermagem Estilo de vida sedentário em jovens adultos. Brasil, 2022

### Proposições

As proposições elaboradas na presente TMA foram:

1. Estímulos pessoais e ambientais estão relacionados com a adoção de comportamentos sedentários;
2. Indivíduos do sexo feminino têm tendência a desenvolver o estilo de vida sedentário;
3. Fatores ligados aos avanços da tecnologia, como internet e smartphones, corroboram com a adoção do estilo de vida sedentário;
4. O estilo de vida sedentário na vida adulta jovem pode ocasionar prejuízos à saúde mental, como depressão, ansiedade e elevados níveis de estresse;
5. O sono insuficiente é comum nos indivíduos sedentários;
6. O estilo de vida sedentário está relacionado com o aumento das chances de desenvolver alterações cardiovasculares em jovens adultos;
7. O estilo de vida sedentário influencia as atividades de vida diária de jovens adultos, principalmente no trabalho e estudo, devido aos prejuízos ocasionados na memória e na função cognitiva;
8. O comportamento sedentário pode ocasionar lombalgia nessa faixa etária;
9. O estilo de vida sedentário em jovens adultos corrobora com o aumento da adiposidade e a diminuição do condicionamento físico.

### Relações de causalidade e a evidência para a prática

As relações causais entre os fatores etiológicos do DE Estilo de Vida Sedentário em jovens adultos foram:

1. Avançar da idade: O processo de envelhecimento ocasiona transições, com destaque para o afastamento de casa e o início da vida universitária ou do emprego. Essas transições geram mudanças físicas, comportamentais e interpessoais, que podem estar ligadas ao comportamento sedentário^(24, 25)^.
2. Desemprego: O desemprego pode ser causador de comportamentos sedentários, devido ao fato de que empregos na juventude, geralmente, são trabalhos que exigem atividades braçais e na posição de pé. Portanto, a realização de atividades físicas regulares é mais comum ^(26)^.
3. Sexo feminino: Por questões culturais e/ou por mudanças decorrentes da puberdade, como o aumento do depósito de gordura, desconforto associado ao ciclo menstrual regular e a redução dos níveis de hemoglobina no sangue, entre outros, o sexo feminino favorece a adoção do estilo de vida sedentário^(27, 28)^.
4. Conhecimento deficiente sobre sedentarismo: Indivíduos que não possuem conhecimento sobre os malefícios do sedentarismo e, por conseguinte, dos benefícios da realização de atividades físicas e da adoção de estilos de vida saudáveis podem desenvolver comportamento sedentário^(29)^.
5. Morar em zona urbana: Residir em zona urbana está associado com a menor realização de atividade física, devido aos estilos de trabalho e rotinas de vida oriundos dos centros urbanos^(30)^.
6. Tempo de tela excessivo: O tempo excessivo de tela (>2h) está relacionado ao sedentarismo, pois geralmente é realizado em posições que envolvem pouco gasto de energia^(26)^.
7. Tempo sentado prolongado: Longos períodos na posição sentada, em média 7h/dia, seja estudando, jogando ou assistindo televisão, são comportamentos sedentários^(31)^.
8. Hábitos pessoais e familiares desfavoráveis para a atividade física: A ausência ou a insuficiência da prática de atividade física e/ou de comportamentos saudáveis em familiares geram maior desinteresse dos jovens adultos na prática de exercícios físicos^(32, 33)^.
9. Poluição e condições ambientais desfavoráveis para a atividade física: Poluição, chuva, calor ou frio intenso podem causar desconforto, fadiga, sonolência, gerando tendência ao comportamento sedentário^(29, 34)^.

Salienta-se o aporte teórico que o enfermeiro necessita para enxergar as reais causas do estilo de vida sedentário em jovens adultos e assim proceder com ações de promoção à saúde considerando todas as particularidades desse público. A TMA surge como uma ferramenta ímpar para a diminuição da lacuna existente entre a teoria e a prática, tornando o processo de enfermagem mais acurado e, portanto, mais efetivo.

## DISCUSSÃO

O estilo de vida sedentário manifesta-se de formas específicas nas diferentes faixas etárias. Esse comportamento de saúde tem forte relação com os avanços tecnológicos. A comodidade e o conforto proporcionados pela modernidade induzem a prática de hábitos sedentários, especialmente entre os jovens adultos^(34)^.

As características pessoais e sociais, como ser estudante, ter educação materna elevada e possuir uma autopercepção de saúde diminuída, mostram-se como atributos essenciais para a presença do diagnóstico de enfermagem Estilo de Vida Sedentário nessa população. Estudos realizados mostram que esses fatores têm forte impacto na adoção da prática de exercício físico. Esses foram relacionados ao ambiente, à cultura e ao sistema socioe-conômico, o que induz à vida sedentária^(35)^.

Ademais, a atividade física menor do que a recomendada e a inatividade física estão fortemente relacionadas às mudanças ocorridas com o avançar da idade, o início da vida adulta, a realização de atividades profissionais ou o desemprego^(36, 37)^. As mudanças ocorridas nesse estágio da vida podem interferir nas ações de cuidado em saúde adotadas pelos jovens adultos.

O conhecimento deficiente dessa população sobre o estilo de vida sedentário pode ser evidenciado no estudo de Campos^(38)^, o qual apontou que os jovens adultos com maiores informações sobre os benefícios da prática regular de exercícios são mais ativos fisicamente, e que isso é inversamente proporcional à falta dessa consciência. Outrossim, o sexo feminino demonstrou forte relação para a adoção do estilo de vida sedentário. Dados que corroboraram com estudos anteriores reafirmam maiores prevalências de sedentarismo nas mulheres nessa faixa etária^(34, 39)^.

Tem-se também que a falta ou a baixa frequência na realização das atividades físicas está relacionada a diversos problemas de saúde entre as mulheres^(40)^. Estudo de coorte realizado no Brasil identificou que as mulheres jovens eram mais inativas e apresentavam maior percentual de gordura corporal^(41)^.

Outro dado importante evidenciado no presente estudo revela que jovens adultos residentes em centros urbanos realizam menos atividades físicas. Estudo desenvolvido com familiares de jovens adultos com sobrepeso e sedentários identificou que 72% dos participantes moram em grandes centros urbanos^(35)^.

Como consequentes clínicos do DE Estilo de Vida Sedentário, teve-se a presença de doenças cardiovasculares e saúde mental prejudicada. Estudo associou o sedentarismo ao aumento dos riscos cardiovasculares em jovens adultos, assim como a alguns fatores psicossociais, como estresse, ansiedade e depressão^(42)^. Outro estudo mostrou que o hábito de praticar exercícios físicos de forma regular melhora os aspectos psicológicos e a saúde mental dos indivíduos^(43)^.

A inaptidão musculoesquelética e a lombalgia são consequentes clínicos do DE em estudo. O condicionamento físico contribui para a capacidade cardiorrespiratória, composição corporal, resistência muscular e postura corporal. Ademais, a realização de atividades físicas regulares, considerando o tipo de exercício, nível e idade, pode contribuir para a prevenção da lombalgia^(44)^. A lombalgia mostra-se presente na população de jovens adultos, relacionada, dentre outras causas, ao estilo de vida sedentário^(45)^.

Ainda, há uma associação entre o tempo de tela excessivo e sono inadequado. Jovens adultos que demandam longos períodos em comportamentos sedentários podem ter sua qualidade do sono diminuída^(46)^. Em adição, a função cognitiva e a memória desse público também podem estar prejudicadas. Pesquisa afirma que a prática de atividades físicas aumenta os níveis de neurotransmissores e melhora o funcionamento cerebral, bem como o corporal, o sistema cognitivo e o processo de memória^(47)^. Portanto, o desempenho intelectual desses jovens pode ser afetado diante da adoção desse comportamento.

Assim, autores encorajam o desenvolvimento de intervenções que estimulem a promoção da saúde dessa população, visando o bem-estar e a qualidade de vida^(34)^. A adoção do estilo de vida sedentário em jovens adultos mostra-se preocupante e com peculiaridades que merecem ser consideradas nas futuras recomendações propostas pela OMS.

### Limitações do estudo

Como limitação do estudo, destaca-se, inicialmente, a restrição da busca aos idiomas inglês, português e espanhol, pois poderiam ter sido publicados artigos em outros idiomas que respondessem à questão de pesquisa. Outra fragilidade foi decorrente da extração dos atributos essenciais, antecedentes e consequentes clínicos realizada somente pelo pesquisador principal, sem o auxílio de outros pesquisadores e/ou de softwares.

Outrossim, tem-se a limitação relacionada à falta de teste da presente teoria na prática clínica. Assim, encoraja-se o desenvolvimento de estudos com o intuito de validar no campo empírico a referida TMA na população de jovens adultos.

### Contribuições para a área

Diante do exposto, o desenvolvimento de TMA contribui para a redução da distância entre teoria e prática e para o fortalecimento da Ciência de Enfermagem. As teorias de médio alcance voltadas para os diagnósticos de enfermagem permitem o conhecimento aprofundado do fenômeno de interesse e, assim, facilitam a identificação precoce dele na prática clínica do enfermeiro.

A TMA do Estilo de Vida Sedentário em jovens adultos pode ser uma ferramenta útil, pois amplia a compreensão do referido fenômeno, seus elementos etiológicos, características clínicas e relações causais. Isso pode contribuir para o raciocínio clínico e o julgamento diagnóstico do enfermeiro no estabelecimento de metas de proteção, promoção e apoio à adoção de estilos de vida saudáveis.

## CONCLUSÕES

A construção teórica identificou três atributos essenciais, a saber: características pessoais e sociais; inatividade física e média de atividade física menor que a recomendada. Também foram identificados 10 antecedentes clínicos e 7 consequentes clínicos. Dentre esses, 3 antecedentes clínicos foram classificados como estímulos focais; 2 como estímulos contextuais; e 5 como estímulos residuais. Em adição, foram desenvolvidos um pictograma, 9 proposições e 11 relações de causalidade e evidências para a prática oriundas das inter-relações entre esses conceitos. Esses passos foram essenciais para a melhor compreensão do diagnóstico estudado.

Dessa forma, a presente construção teórica é oportuna e produz ferramentas que podem auxiliar na promoção da saúde e qualidade de vida dessa população específica. Uma vez que favorecerá a identificação precoce pelo enfermeiro do fenômeno Estilo de Vida Sedentário em jovens adultos, com destaque para os seguintes fatores etiológicos: hábitos pessoais e familiares desfavoráveis para a atividade física; tempo de tela excessivo; tempo sentado prolongado; e conhecimento deficiente sobre sedentarismo. Esses fatores etiológicos foram destacados, pois estão mais diretamente relacionados à tomada de decisão independente do enfermeiro e podem, ao serem manipulados, promover comportamentos saudáveis e aumento do conforto.

Estudos teóricos sobre os diagnósticos de enfermagem devem ser realizados com o objetivo de tornar o cuidado de enfermagem mais eficaz, individualizado e de maior qualidade, de modo a fortalecer a Ciência da Enfermagem.
